# Future behaviours decision-making regarding travel avoidance during COVID-19 outbreaks

**DOI:** 10.1038/s41598-022-24323-1

**Published:** 2022-11-17

**Authors:** Koichi Ito, Shunsuke Kanemitsu, Ryusuke Kimura, Ryosuke Omori

**Affiliations:** 1 Division of Bioinformatics, International Institute for Zoonosis Control, Hokkaido University, Sapporo, Hokkaido 001-0020 Japan; 2 Data Solution Unit 2 (Marriage and Family/Automobile Business/Travel), Data Management and Planning Office, Product Development Management Office, Recruit Co., Ltd, Chiyoda-ku, Tokyo, 100-6640 Japan; 3 SaaS Data Solution Unit, Data Management & Planning Office, Product Development Management Office, Recruit Co., Ltd, Chiyoda-ku, Tokyo, 100-6640 Japan

**Keywords:** Data mining, Human behaviour, Epidemiology

## Abstract

Human behavioural changes are poorly understood, and this limitation has been a serious obstacle to epidemic forecasting. It is generally understood that people change their respective behaviours to reduce the risk of infection in response to the status of an epidemic or government interventions. We must first identify the factors that lead to such decision-making to predict these changes. However, due to an absence of a method to observe decision-making for future behaviour, understanding the behavioural responses to disease is limited. Here, we show that accommodation reservation data could reveal the decision-making process that underpins behavioural changes, travel avoidance, for reducing the risk of COVID-19 infections. We found that the motivation to avoid travel with respect to only short-term future behaviours dynamically varied and was associated with the outbreak status and/or the interventions of the government. Our developed method can quantitatively measure and predict a large-scale population’s behaviour to determine the future risk of COVID-19 infections. These findings enable us to better understand behavioural changes in response to disease spread, and thus, contribute to the development of reliable long-term forecasting of disease spread.

## Introduction

The emergence of COVID-19 has reaffirmed the need to control the spread of infectious diseases through efficient monitoring and forecasting. However, the role of epidemic forecasting during the spread of COVID-19 was mostly limited. In fact, most reliable forecasting was focused on predicting new cases over subsequent weeks, whereas long-term forecasts, especially to predict peaks and rebounds in incidences, were deemed challenging^1–3^. There are many reasons why long-term forecasting is said to be difficult. For instance, the ecology and evolution of emerging infectious diseases remains largely unknown; how the immune system of a host responds to a new disease is poorly understood and as is the host’s change in behaviours^2,4,5^.

In this article, we are mostly concerned about people’s behavioural responses to epidemic events preventing disease forecasting^2,6–8^. Changes in behaviours have long been observed during epidemics. These include precautionary measures that were adopted during the severe acute respiratory syndrome pandemic^9^; reduced public transport use, rescheduling travel plans, or cancellation of commercial flights^10^; mask-wearing and more frequent hand sanitising^11^ during H1N1 pandemic. Additionally, avoidance of unsafe traditional burials during Ebola outbreaks^12^; and the numerous measures, such as reduced human mobility^13^, that were taken during the current COVID-19 outbreak^14^. Such behavioural responses are known to help suppress the spread of an infectious disease^7,8,15^, which in turn may also cause additional behavioural responses. In such a case, the effect of the behavioural response on the spread of disease becomes more crucial and complex, which makes it necessary to predict future behaviours for the long-term forecasting of infectious outbreaks.

To predict human behaviours, we must first understand the decision-making involved in behavioural responses. The kinds of observations, however, are difficult because we can usually observe only realised behaviours because of such decision-making. For example, large-scale human mobility data from mobile phones^16,17^, smart cards^18^, and/or social network services^19^ have been used to estimate the spatial and temporal spread of infectious diseases^17,20–25^ or evaluate the effect of government interventions^26–30^. However, human behaviours may be decided based on both the present situation as well as the past because we often need planning, appointment, or reservation in advance of the behaviour. Thus, human mobility data can show only realised behaviours, but not the timing of the decision for the observed behavioural changes. This makes it unsatisfactory to identify factors that influence decision-making from only mobility data.

We need a fundamentally different approach to observe the decision-making of human behaviours in response to COVID-19. One possibility is to use accommodation reservation data. It is generally believed that travel can increase the risk of infectious spread^31,32^. In fact, travel restrictions were one of the earliest government-mandated responses to COVID-19 in Japan^33^. Thus, accommodation reservations form an interesting dataset that reflect behavioural changes in response to government interventions or outbreak status^11,31^. Importantly, making new reservations or cancelling existing ones are decision-making events for future behaviours and are observed as a fall in new reservations or an incremental increase in cancellations. In other words, accommodation reservation data allow us to quantitatively evaluate the decision-making of a large-scale population for future risk reduction behaviours.

## Materials and methods

### Data

The accommodation reservation dataset excluding personally identifiable information was obtained from jalan.net (https://www.jalan.net/), one of the largest online travel agents in Japan^34^. All reservation records for accommodations located in four prefectures, Miyagi, Aichi, Osaka, and Fukuoka, from 1 January 2016 to 31 December 2021 were enrolled in the analysis. To avoid bias from spatial heterogeneity, we chose the prefectures showing the largest population in each region of Japan in 2020^35^ (see Fig. S2). The number of accommodations located in the four prefectures is 2065 (318 for Miyagi, 543 for Aichi, 629 for Osaka, and 575 for Fukuoka, counted on the jalan.net website on 8 February 2022), which comprised 47.8% of the accommodations reported by Japan Tourism Agency (2021)^36^ (46.2% for Miyagi, 55.9% for Aichi, 42.2% for Osaka, and 47.3% for Fukuoka). Each reservation record contained the reserved date, accommodation date, and cancelled date if the reservation was cancelled. Since reservation records for a stay more than 1 year ahead are rare (less than 0.0015% of all records), only reservation records for a stay within 365 days were used for the analyses. The number of newly reported COVID-19 cases in Japan was obtained from the open dataset provided by the Ministry of Health, Labour and Welfare of Japan^37^. The date at which the government declared a state of emergency was obtained from Cabinet Secretariats^38^.

### Model

Depending on the spread of the epidemic or government’s intervention, the degree of motivation for avoiding travel can be varied. We defined such motivations for a certain future period at each period as the ‘travel avoidance level’. The higher the travel avoidance level, the higher the probability of postponing accommodation reservations *P*_*R*_ or cancelling the existing reservations *P*_*C*_. The influence of travel avoidance level might differ between *P*_*R*_ and* P*_*C*_; for example, either might show a more gradual change or appear only under higher travel avoidance levels. To allow for such differences in responses, we assumed that these probabilities owing to travel avoidance levels are represented by sigmoid functions, that is,

1a $$ P_{R} = \frac{1}{{1 + \exp (a({\text{logit}}(\lambda_{t,x} ) - b))}} $$

and

1b $$ P_{C} = \frac{1}{{1 + \exp (c({\text{logit}}(\lambda_{t,x} ) - d))}} $$

where $\lambda_{t,x}$ is the travel avoidance level at time *t* for the travel *x* days ahead, and *a*, *b* or *c*, *d* are coefficients determining the slope and the threshold of the sigmoid functions. *logit* is the logit function, that is,

2 $$ {\text{logit}}(\lambda ) = \ln \left( {\frac{\lambda }{1 - \lambda }} \right) $$

The expected number of the accommodation reservations for the stay on *x* days ahead at time *t* is

3a $$ \begin{aligned} R_{t,x} & = \overline{R}_{x} (1 - P_{R} ) \\ & = \overline{R}_{x} \left( {1 - \frac{1}{{1 + \exp (a({\text{logit}}(\lambda_{t,x} ) - b))}}} \right) \\ \end{aligned} $$

where $\overline{R}_{x}$ is the baseline occurrence frequency of the reservation event for the stay on *x* days ahead. When $\lambda_{t,x}$ = 0, the expected number of accommodation reservations becomes equal to the baseline $\overline{R}_{x}$, and when $\lambda_{t,x}$ = 1, no new reservation occurs. Similarly, the cancellation probability of the existing reservations per day is represented as

3b $$ \begin{aligned} C_{t,x,y} & = \overline{C}_{x,y} + (1 - \overline{C}_{x,y} )P_{C} \\ & = \overline{C}_{x,y} + (1 - \overline{C}_{x,y} )\frac{1}{{1 + \exp (c({\text{logit}}(\lambda_{t,x} ) - d))}} \\ \end{aligned} $$

where $\overline{C}_{x,y}$ is the baseline cancellation probability of the reservation, which is reserved on *y* days ahead of the stay and cancelled on *x* days ahead of the stay. When $\lambda_{t,x}$ = 0, the expected number of the cancellation probability becomes equal to the baseline $\overline{C}_{x,y}$, and when $\lambda_{t,x}$ = 1, all existing reservations are cancelled.

To reduce the number of parameters for the estimation, we rewrite Eqs. (3a) and (3b) by the parameter transformation as follows:

4a $$ R_{t,x} = \overline{R}_{x} \left( {1 - \frac{1}{{1 + \exp ({\text{logit}}(\lambda^{\prime}_{t,x} ))}}} \right) $$

and

4b $$ C_{t,x,y} = \overline{C}_{x,y} + (1 - \overline{C}_{x,y} )\frac{1}{{1 + \exp (c^{\prime}({\text{logit}}(\lambda^{\prime}_{t,x} ) - d^{\prime}))}} $$

where

5a $$ \lambda^{\prime}_{t,x} = \frac{{\exp \left[ {a\left( {\ln \left( {\frac{{\lambda_{t,x} }}{{1 - \lambda_{t,x} }}} \right) - b} \right)} \right]}}{{1 + \exp \left[ {a\left( {\ln \left( {\frac{{\lambda_{t,x} }}{{1 - \lambda_{t,x} }}} \right) - b} \right)} \right]}} $$

5b $$ c^{\prime} = \frac{c}{a}\quad {\text{and}} $$

5c $$ d^{\prime} = a(b - d) $$

This parameter transformation does not qualitatively change the influence of the levels of travel avoidance. In other words, the change of the probability of postponing accommodation reservations or cancelling existing reservations owing to the motivation of avoiding travel could be aggregated into $\lambda^{\prime}_{t,x} ,\;c^{\prime},\;{\text{and}}\;d^{\prime}$.

### Estimation

$\overline{R}_{x}$ and $\overline{C}_{x,y}$ are derived by calculating the mean weekly reservation frequency and cancellation probability before the emergence of COVID-19 from the accommodation reservation data between 1 January 2016 and 31 December 2019. Following the previous studies of the reservation behaviours, we assumed that the observed new reservation numbers at each week are following the Poisson distribution whose expected occurrence number is Eq. (4a), and the observed cancellation numbers are following the binomial distribution whose occurrence probability is Eq. (4b) and trial number is the number of ‘survived’ (not cancelled yet) reservation^39^. Based on these assumptions, the levels of travel avoidance at week *t* for *x* days ahead, $\lambda^{\prime}_{t,x}$, and the coefficients of cancellation in response to the travel avoidance levels, *c*′ and *d*′, are estimated by maximum likelihood estimation. Likelihood function is given by

6 $$ L(c^{\prime},d^{\prime},\lambda^{\prime}_{t,x} ) = \prod\nolimits_{t} {\prod\nolimits_{x} {{\text{pmf}}({\text{poisson}}(R_{t,x} ),R_{t,x,Data} ) \times } } \prod\nolimits_{t} {\prod\nolimits_{x} {\prod\nolimits_{y} {{\text{pmf}}({\text{Bin}}(C_{t,x,y} ,\;N_{t,x,y,Data} ),M_{t,x,y,Data} )} } } , $$

where $R_{t,x,Data}$ is the observed number of accommodation reservations for the stay on *x* days ahead at week *t*. $N_{t,x,y,Data}$ is the observed number of the survived reservations on *x* days ahead of the stay at week *t*, which was the reservation on *y* days ahead of the stay; and $M_{t,x,y,Data}$ is the observed number of cancellations on *x* days ahead of the stay at week *t*, which was the reservation on *y* days ahead of the stay, respectively. Then, *pmf*(*poisson*(*E*),*x*) and *pmf*(*Bin*(*n*,*p*),*x*) denote the probability mass function of the Poisson and binomial distribution when the expected number of observed events is *E*, the number of observed events is *x*, the trial number is *n*, and the probability that an event occurs is *p*.

The estimation of $\lambda^{\prime}_{t,x}$ and coefficients {*c*′, *d*′} maximising the likelihood function *L* was done as follows. The maximum likelihood estimate of $\lambda^{\prime}_{t,x}$ is referred to as $\lambda_{t,x}^{*}$. To this end, first, for the given coefficients pair of {*c*′, *d*′}, $\lambda^{\prime}_{t,x}$ maximising the likelihood, as described in Eq. (6), ${\lambda }_{t,x}^{{{\prime\prime}}}({c}^{{\prime}},{d}^{{\prime}})$, is computed using Brent’s method. Next, the coefficients pair {${c}^{{\prime}},{d}^{{\prime}}$} maximising $L\left({c}^{{\prime}},{d}^{{\prime}},{\lambda }_{t,x}^{{{\prime\prime}}}({c}^{{\prime}},{d}^{{\prime}})\right)$, {${c}^{*},{d}^{*}$}, is obtained using the Nelder–Mead method. Therefore, ${\lambda }_{t,x}^{*}$ is given by ${\lambda }_{t,x}^{{{\prime\prime}}}({c}^{*},{d}^{*})$. ${\lambda }_{t,x}^{*}$ was smoothed by the locally weighted smoothing method along $x$ days direction. The estimated ${\lambda }_{t,x}^{*}$ before applying the locally weighted smoothing method and the coefficients {${c}^{*},{d}^{*}$} are shown on Supplementary File S1.

### Travel avoidance levels against COVID-19

Reservation and cancellation are associated with factors other than COVID-19. To extract travel avoidance levels against COVID-19 specifically, we compared $\lambda_{t,x}^{*}$ between before and after the emergence of COVID-19 assuming factors other than COVID-19 were similar even after the emergence of COVID-19. We measured the travel avoidance levels against COVID-19, $\hat{\lambda }_{t,x}$, as follows:

7 $$ \hat{\lambda }_{t,x} = \frac{{\lambda_{t,x}^{*} - \overline{\lambda }_{x} }}{{1 - \overline{\lambda }_{x} }} $$

where $$\overline{\lambda }_{x}$$ is the mean measured travel avoidance level for *x* days ahead before the outbreaks of COVID-19 (between 1 January 2016 and 31 December 2019). ‘Travel avoidance levels’ in the main text refers to the travel avoidance levels against COVID-19, that is, $$\hat{\lambda }_{t,x}$$$$.$$

### Analysis

For the statistical test of significance of differences in the responses of travel avoidance levels in the short- and long-term future, we compared the two variances of $\hat{\lambda }_{t,x}$ after the emergence of COVID-19 with $x<90$ and $\ge 90$ days by Levene’s test. The correlation of $\hat{\lambda }_{t,x}$ with the number of reported cases is calculated using Spearman’s rank correlation coefficient, Kendall’s rank correlation coefficient, and maximal information coefficient. All analyses were performed in R version 4.0.4 with RStudio interface version 1.4.1717, R package ‘Rcpp’ version 1.0.7, ‘tidyverse’ version 1.3.1, and GNU compiler collection version 11.2.0. Levene’s tests were performed by R package ‘lawstat’ version 3.4. The maximal information coefficients were derived by R package ‘minerva’ version 1.5.10. All figures were made using R package ‘ggplot2’ version 3.3.5 and ‘RColorBrewer’ version 1.1-2.

## Results

In this study, our aim is to measure decision-making for travel avoidance under COVID-19 based on accommodation reservation data. To simplify, government intervention and/or an increase in infectious spread will motivate people to change future behaviours to lessen the risk of contracting a disease. We observe this ‘change’ through accommodation reservation data showing the reduction in new reservations or increase in cancellations. We model these travel avoidances and compare them with real accommodation reservation data to measure the levels of the travel avoidance for a certain-term future at each week.

**Figure 1:**
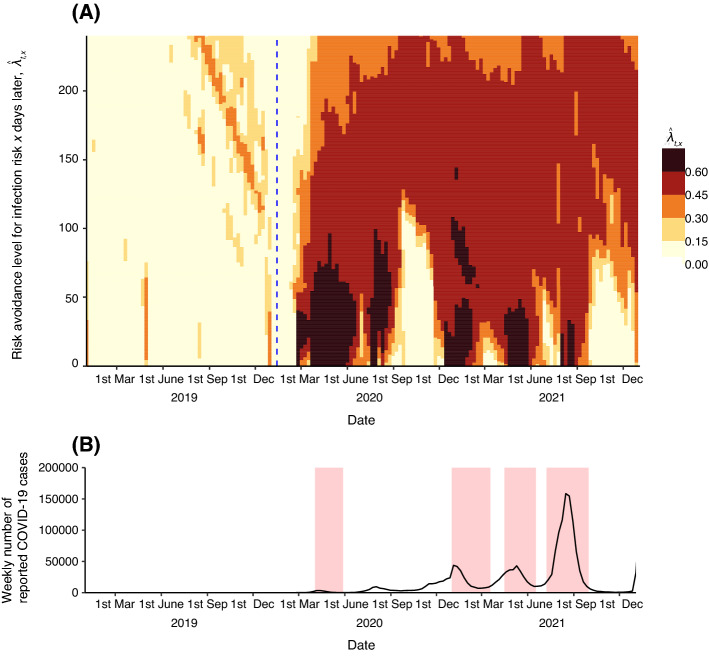
Time evolution of human behavioural response to COVID-19. (**A**) Time evolution of travel avoidance level for the travel *x* days later at time *t*, $\hat{\lambda }_{t,x}$. The colours show the estimated values of $\hat{\lambda }_{t,x}$. Vertical dashed line shows the report timing of the first COVID-19 case in Japan. (**B**) Time evolution of weekly number of COVID-cases in Japan. Filled pale red colour squares show the timing when the Japan government declared a state of emergency.

Figure 1A shows the evaluated travel avoidance levels in response to COVID-19. In 2019, the travel avoidance levels were low at any point of time in the future (the mean travel avoidance levels before the COVID-19 outbreaks were normalised to zero; the 5–95 percentile range is [− 0.481, 0.408]). This tendency continued even after the first case of COVID-19 was confirmed in Japan on 16 January 2020 (indicated by a blue dashed line; see also Fig. S1A). At the end of the February 2020, the travel avoidance levels rapidly rose and became clearly high after the first declaration of a state of emergency by the Japanese government after 4 April 2020 (the mean travel avoidance level is 0.430; the 5–95 percentile range is [0.026, 0.621]).

**Figure 2:**
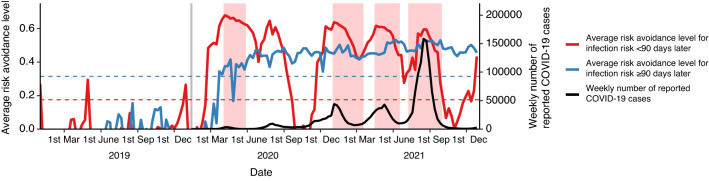
Comparison of travel avoidance levels by short-term and long-term prediction. True lines show the average travel avoidance level for the travel < 90 days later (red) and that for the travel ≥ 90 days later (blue), respectively. Black true line shows weekly number of reported COVID-19 cases in all of Japan. Dashed lines show 95 percentiles of the average travel avoidance level for the travel < 90 days later (red) and ≥ 90 days later (blue). Grey vertical line shows the timing of first the COVID-19 case in Japan.

In terms of sensitivity to the change in the COVID-19 outbreak status, we observed a significant difference between the responses of the travel avoidance levels for the short- and long-term future (Levene’s test, p < 0.05) (blue and red line in Fig. 2). The travel avoidance levels for the first 3 months (red curve in Fig. 2) rapidly grew between the first report of COVID-19 case in Japan (grey line in Fig. 2) and the first declaration of the emergency (pale red areas in Fig. 2). Whereas those for more than 3 months ahead (blue curve in Fig. 2) were still low (see Fig. S1B). At the first declaration of emergency, the travel avoidance levels for more than 3 months ahead also heightened (see Fig. S1C). After, although the travel avoidance levels for next 3 months dynamically varied in response to the outbreak status or government interventions, the travel avoidance levels for more than 3 months ahead remained high regardless of the situation (Fig. 2). After April 2020, the observed travel avoidance levels can be qualitatively categorised into two patterns. First, the travel avoidance levels were high at any future time point when the number of reported cases of COVID-19 were high and/or when the government declared a state of emergency (see Fig. S1E). Second, the travel avoidance levels for next few months were low, whereas those for more than 3 months ahead remained high when the reported new cases were low and there was no government intervention (see Fig. S1D,F).

**Figure 3:**
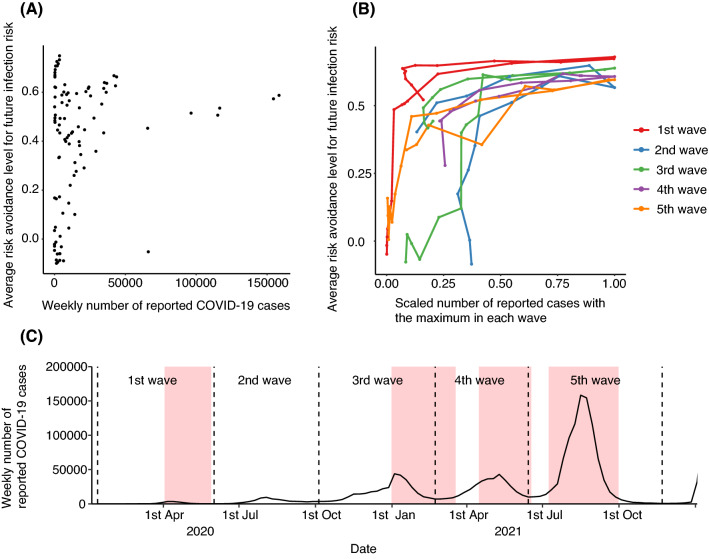
Human behavioural response against COVID-19 with the number of reported cases. (**A**) Weak correlation relation between the travel avoidance level and the number of reported COVID-19 cases. Vertical axis shows the average travel avoidance level for the travel from 0 to 365 days later. Horizontal axis shows the number of reported COVID-19 cases in all of Japan. (**B**) Stratification of time-series of reported cases by the wave of epidemic improves the correlation with the travel avoidance level. Vertical axis shows the average travel avoidance level for the travel from 0 to 365 days later. Horizontal axis shows the scaled number of reported cases with the maximum number of reported cases in each wave of COVID-19 in all of Japan, which is equal to unity at the peak of each wave. The colours denote the waves of COVID-19 in Japan. (**C**) The definition of waves of COVID-19 in Japan. Filled red colour squares show the timings when the Japanese government declared a state of emergency.

Although the travel avoidance levels for the next 3 months seemed to synchronously vary with the outbreak status (Fig. 2), the correlation between the travel avoidance levels and the weekly number of reported cases was weak (Spearman’s rank correlation coefficient is 0.192; see Fig. 3A and Table S1). However, if the COVID-19 pandemic in Japan is classified into five waves (Fig. 3C), the travel avoidance levels for the next 3 months become strongly correlated with the number of reported cases (Spearman’s rank correlation coefficients at each wave are 0.902, 0.654, 0.849, 0.932, and 0.926; see Fig. 3B and Table S1). The correlation coefficients for the second wave were relatively weaker than for the other waves (Table S1). For the first wave (from 16 January to 21 June 2020), the travel avoidance levels for next 3 months were remarkably high compared with the other four waves (Fig. 3B). The maximum of the weekly reported-case numbers at each wave increased in later waves (Fig. 3C), indicating that the response of travel avoidance levels to the absolute number of reported cases weakened in later waves.

We also measured the travel avoidance levels in response to COVID-19 separately at each of four prefectures targeted in this study, namely Miyagi, Aichi, Osaka, and Fukuoka (see Fig. S2). We found that (i) the travel avoidance levels drastically increased after the emergence of COVID-19; (ii) the travel avoidance levels for the short-term future varied with the change in outbreak status, whereas those for the long-term future remained high; and (iii) there was high correlation of the travel avoidance levels with the number of reported cases stratified by the waves of COVID-19. These findings were robust between the four prefectures (see Fig. S3) despite their geographical distances.

## Discussion

We applied accommodation reservation data to evaluate decision-making on future behaviours for reducing the risk of infection. Our analysis clearly shows the dynamics of the travel avoidance levels of the Japanese with the progress of the outbreak. After the emergence of COVID-19 in Japan, travel avoidance levels for the next 3 months dynamically changed in respond to the outbreak status and government interventions. We see that the travel avoidance levels for more than 3 months ahead remained high after the outbreak in Japan. These results reveal how people estimated the future risk of infections and changed their behaviours.

Our analyses highlight the factors that influenced Japanese people’s decision-making to avoid travel. For example, even after the first report of COVID-19 case in Japan, the travel avoidance levels were similar to the levels before the emergence of COVID-19 during the following weeks (Fig. S1A). It may be that the reports of COVID-19 infections were limited at the very early stage of the outbreak. Indeed, rapid growth of travel avoidance levels was observed around the time a task force was established by the Ministry of Health, Labour and Welfare to contain COVID-19 clusters by 25 February 2020^40^. Thus, the incremental reports of COVID-19 seemed to have triggered an equivalent increment in travel avoidance levels. Similarly, in March 2020, the travel avoidance levels for the next 3 months grew, whereas that in proceeding more than 3 months remained low (Fig. S1B). Thus, people predicted that the outbreak could be over within 3 months.

The correlation between the travel avoidance levels for the next 3 months and the number of reported cases was weak for the entire COVID-19 outbreak period in Japan, whereas the correlation was strong in the analyses for each wave. This result suggests that people evaluated the risk based not on the number of reported cases itself but based on a comparison of the current number of reported cases in the recent trend. Considering that the maximum of weekly reported-case numbers at each wave was higher in the later wave, the response to the absolute number of reported cases weakened in the later wave, indicating habituation to the absolute number of reported cases^41,42^. The correlation between the travel avoidance levels for the next 3 months and the number of reported cases was also weaker for the second wave compared with the other waves; probably because a state of emergency was not declared for the second wave.

Interestingly, after April 2020, the travel avoidance levels for more than 3 months ahead remained high, regardless of the reduction in the number of reported cases or the relaxation of government restrictions. In this period, it is possible that people’s confidence in their own future predictions grew; however, there still existed difficulty in making predictions more than 3 months ahead owing to the high uncertainty. Thus, the factors causing high level of travel avoidance might be different for the short- and long-term future; that is, the travel avoidance behaviours for the short-term future are determined by people’s own future prediction, whereas those for the long-term future were constant because of higher uncertainty.

We successfully showed that risk reduction of future behaviours can be measured using the accommodation reservation data. These data have two essential differences from the typical human mobility data. First, they contain information about two different events, namely new reservations and cancellations of existing reservations. We cannot estimate the travel avoidance levels from only one because it is impossible to estimate both the travel avoidance levels and the behavioural response to the travel avoidance levels (in our cases coefficients of sigmoid functions) simultaneously. Since these two events are mutually independent, we can estimate the travel avoidance levels, which then influence the occurrence of both events simultaneously. Second, these data contain information about future behaviour. The effect of human behaviour on the disease spread has been examined using various data sources such as human mobility data or social network services^19^. However, such data contain only the past or mostly real-time information. In contrast, accommodation reservation data deal with decision-making for future behaviours at a specific time, and therefore, allow us to forecast future behaviour.

Our method has some clear advantages over prior methods for evaluation of behavioural responses. First, our method can quantitatively evaluate the decision-making of large-scale populations with little effort. Second, the accommodation reservation data are a direct observation of decision-making, and thus, free from response biases, which are common in the assessment of attitudes in questionnaires^43^. Third, this method can be applicable to any other accommodation reservation data regardless of country, periods, and trigger event (e.g. it can be applied to gauge the responses to natural disasters or political conflicts). A similar method could be applied to reservations to sports facilities, restaurants, or health-related clinics.

Although our study successfully revealed the behavioural changes in response to COVID-19, further studies are required to better understand these changes. First, the causality of the detected change in travel avoidance levels should be examined. For example, we showed that travel avoidance levels for the next 3 months varied with the number of new reported cases (see Fig. 1A,B); although we did not analyse the causality between them. Importantly, the behavioural changes might be not only the result of the spontaneous decision-making of customers but also the forced changes, for example, travel restrictions owing to government intervention, suspension of transportation methods to get to the accommodation, or the temporary accommodation closure for business reasons. The effectiveness of government interventions could be evaluated by focusing on the drastic change in travel avoidance levels and measuring the types of information or events that have a critical influence on human behaviour decision-making.

Second, the influence of our estimated travel avoidance level on other types of behavioural changes besides accommodation reservation is unclear. For instance, a comparison with precautionary measures against infection adopted or the avoidance of public transport, which have been reported during COVID-19 outbreaks^13,14^, can reveal the change in wider variations of behaviours to understand precise human response to emerging outbreaks of infectious disease. For such further applications, it is important that the reservation dataset contains (i) not only the reservations that were kept (when the customer actually visited the place) but also cancelled reservations, (ii) a broad and large enough sample so that the recorded behavioral changes can be approximated as the tendencies of the overall society, and (iii) reservation records before the emergence of the infectious disease in question.

In conclusion, we demonstrated that the decision-making for future behaviours to avoid travels for reducing the risk of contracting COVID-19 could be observed from accommodation reservation data. This method can quantitatively measure a large-scale population’s predictions for the future risk of contracting COVID-19. The motivation of risk reduction for short-term future behaviours dynamically varied and was associated with the outbreak status and/or government interventions. Our results provide essential information for the prediction of human responses to an epidemic.

## Supplementary Information

Supplementary Information 1.

Supplementary Information 2.

Supplementary Information 3.

## Data Availability

The data that support the findings of this study are available from Recruit Co., Ltd. but restrictions apply to the availability of these data, which were used under license for the current study, and so are not publicly available. Data are however available from the authors upon reasonable request and with permission of Recruit Co., Ltd. The scripts for the analysis of the maximum likelihood estimation are available from the corresponding author on reasonable request.
