# Supplementary material for: Future behaviours decision-making regarding travel avoidance during COVID-19 outbreaks
Source: Sci Rep. 2022 Nov 17;12:19780. doi: 10.1038/s41598-022-24323-1 (PMC9671889; doi:10.1038/s41598-022-24323-1)
Supplement: Supplementary file 1 — Supplementary Information 1. [file 41598_2022_24323_MOESM1_ESM.docx]

**Supplementary Information for**

Future behaviours decision-making regarding travel avoidance during COVID-19 outbreaks.

Koichi Ito^1^, Shunsuke Kanemitsu^2^, Ryusuke Kimura^3^, Ryosuke Omori^1^*

^1^Division of Bioinformatics, International Institute for Zoonosis Control, Hokkaido University; Sapporo, Hokkaido, 001-0020, Japan.

^2^Data Solution Unit 2(Marriage & Family/Automobile Business/Travel), Data Management & Planning Office, Product Development Management Office, Recruit Co., Ltd; Chiyoda-ku, Tokyo, 100-6640, Japan

^3^SaaS Data Solution Unit, Data Management & Planning Office, Product Development Management Office, Recruit Co., Ltd; Chiyoda-ku, Tokyo, 100-6640, Japan

*Ryosuke Omori

**Email:**  [omori@czc.hokudai.ac.jp](mailto:xxxxx@xxxx.xxx)

**Figure S1.** Snapshots of travel avoidance level for the travel in the future at six time points. (**A**) before COVID-19, (**B**) the early phase of COVID-19, (**C**) around the peak of the first wave, (**D**) between the second and third waves, (**E**) around the peak of the third wave, (**F**) between the fourth and fifth waves, and (**G**) locations of time points on the epidemic curve of COVID-19 in Japan (a-f in panel (**G**) are correspond to the time points of panel (**A**)-(**F**)).

**Figure S2.** The locations of Miyagi, Aichi, Osaka, and Fukuoka prefectures.

**Figure S3.** Time evolution of human response against COVID-19 in four prefectures. Travel avoidance level for the travel $x$ days later at time $t$, $\hat{\lambda}_{t,x}$ in (**A**) Miyagi, (**B**) Aichi, (**C**) Osaka, (**D**) Fukuoka prefecture. The colours show the estimated values of $\hat{\lambda}_{t,x}$. (**E**) Time evolution of weekly number of COVID-cases in Japan. Filled red-coloured squares show the timing when the Japan government declared a state of emergency.

|  |  | Waves | | | | |
| --- | --- | --- | --- | --- | --- | --- |
|  | All | 1st | 2nd | 3rd | 4th | 5th |
| Start Date | 2020-01-16 | 2020-01-16 | 2020-06-22 | 2020-10-05 | 2021-03-15 | 2021-06-28 |
| End Date | 2021-12-31 | 2020-06-21 | 2020-10-04 | 2021-03-14 | 2021-06-27 | 2021-11-21 |
| Spearman rank correlation coefficient | 0.192 | 0.904 | 0.654 | 0.849 | 0.932 | 0.926 |
| Kendall rank correlation coefficient | 0.158 | 0.782 | 0.467 | 0.692 | 0.79 | 0.79 |
| Maximal information coefficient | 0.432 | 0.776 | 0.538 | 0.785 | 0.706 | 0.998 |

**Table S1.** The correlation between the travel avoidance level and outbreak status stratified by the five waves of COVID-19 pandemic in Japan. The correlation between the average travel avoidance level for the travel from 0 day to 365 days later and the relative number of reported cases to the maximum number of reported cases in each wave of COVID-19 in Japan was calculated using Spearman rank correlation coefficient, Kendall rank correlation coefficient, and maximal information coefficient.

**File S1**

File S1 contain the following six csv files. ***Parameters.csv*** shows the estimated model parameter values $c$^*^, $d^{*}$ in Miyagi, Aichi, Osaka, Fukuoka and All of four prefectures. ***Miyagi_lambda.csv***, ***Aichi_lambda.csv***, ***Osaka_lambda.csv***, ***Fukuoka_lambda.csv*** and ***All_lambda.csv*** shows the estimated model parameter values $\lambda_{t,x}^{*}$ on Miyagi, Aichi, Osaka, Fukuoka and All of four prefectures. First and second columns shows the start and end date of the estimated $\lambda_{t,x}^{*}$ (i.e., range of term *t*), and the following other columns named “day*x*” shows the estimated $\lambda_{t,x}^{*}$ values on *x* days ahead.

**File S2**

File S2 contain the source code for the analysis and figure plotting. See README.md for the detail.
